# Proteins S100A8 and S100A9 are potential biomarkers for renal cell carcinoma in the early stages: Results from a proteomic study integrated with bioinformatics analysis

**DOI:** 10.3892/mmr.2015.3321

**Published:** 2015-02-09

**Authors:** LIMIN ZHANG, HAOWEN JIANG, GANG XU, HUI WEN, BIN GU, JUN LIU, SHANGHUA MAO, RONG NA, YAN JING, QIANG DING, YUANFANG ZHANG

**Affiliations:** Department of Urology, Huashan Hospital, Fudan University, Shanghai 200040, P.R. China

**Keywords:** renal cell carcinoma, serum biomarker, protein S100A8, protein S100A9, bioinformatics

## Abstract

In order to investigate the two members of the EF-hand Ca^2+^ binding protein S100 family, S100A8 and S100A9, in renal cell carcinoma (RCC), serum samples were collected from patients with RCC, transitional cell carcinoma in the kidney, benign renal masses and normal controls. The samples were analyzed by isobaric tags for relative and absolute quantification technology to identify the differential expression of S100A8 and S100A9 in the respective groups. Hierarchical clustering analysis was then conducted for the samples and the relevant selected gene. The cross-platform analysis for the external validation was performed by means of The Cancer Genome Atlas database, containing the gene/microRNA expression pattern and clinical information of patients with RCC. Immunohistochemical staining was used to verify the expression of S100A8 and S100A9 in the four groups. As a result, serum and mRNA expression levels of S100A8 and S100A9 were found to be upregulated in patients with RCC compared with the other three groups, which was consistent with the result of the upregulated expression of mRNA levels in RCC tissue. The overexpression of S100A8 and S100A9 in cancer cells was also confirmed by immunohistochemistry. In addition, bioinformatics revealed that let-7, a microRNA formerly identified as an inhibiting factor of RCC was downregulated in RCC, which contrasted with S100A8. It was also complementary to the sequence at the 3′ untranslated region terminal of S100A8. Therefore, indicating that S100A8 and S100A9 may serve as biomarkers for the detection of RCC.

## Introduction

Patients with renal cell carcinoma (RCC) often present with few signs, symptoms or laboratory abnormalities and are frequently (~30%) diagnosed at the metastatic stage, when the prospects for curing the disease are poor, with a 9% five-year survival rate (1).

According to evidence from clinical and scientific studies, immune-mediated mechanisms are involved in the growth and progression of RCC (2,3). For instance, RCC can evoke an immune response, occasionally leading to spontaneous and significant remission (2). Clinical benefits achieved with systemic cytokine therapy, including interleukin-2 and interferon-α, provide direct evidence for the development of immune responses against RCC (3).

The proteins S100A8 and S100A9 are EF-hand Ca^2+^ binding proteins belonging to the S100 family, which are abundant in the cytosol of phagocytes and are critical in numerous cellular processes, including motility and danger signaling by interacting with and modulating the activity of target proteins (4–6). S100A9 exists with S100A8 as homodimers, preferentially forming functional anti-parallel heterodimers of S100A8/A9 (4). They are constitutively expressed in myeloid cells, including granulocytes and monocytes, accounting for multiple functions in the innate immune system (7). The expression levels of S100A8 or the heterodimer, S100A8/A9, are increased in tumor cells in multiple types of cancer, including gastric (8), colon (9) and pancreatic cancer (8), but rarely in RCC.

In the present study, the serum levels of S100A8/A9 was compared in different groups of patients with RCC, transitional cell carcinoma (TCC) in the kidney, benign lesions in the kidney and normal controls. In addition, integrated analysis of bioinformatics for the protein and mRNA of S100A8/A9 was performed using The Cancer Genome Atlas (TCGA) RCC database. Furthermore, an immunohistochemistry assessment was performed in tissue samples from RCC, TCC, renal cysts and normal kidney for validation.

## Materials and methods

### Detection of differential expression in serum levels of S100A8 and S100A9 by isobaric tags for relative and absolute quantification (iTRAQ) technology

Serum samples were obtained from 29 patients with RCC, 24 patients with benign mass lesion in the kidneys, 28 patients with other types of urological tumor (20 cases with TCC and 8 cases with prostate cancer or male genital tumors) and 18 healthy individuals used as the control. Patients with accompanying kidney diseases, cardiovascular disorders and other types of cancer, with the exception of urological tumors, were excluded. The study was approved by the ethics committee of Huashan Hospital, Fudan University (Shanghai, China) and written informed consent was provided prior to the assessment. All RCC patients were at the T1a stage, confirmed by radiological evaluation and pathological assessment of the surgical specimen. Sera from the four groups mentioned previously were pooled, with the abbreviation R for RCC, C for benign lesions in kidneys, M for other urological tumors and H for non-cancer control. Subsequently, the iTRAQ-labeled samples were pooled and fractionated by strong-cation exchange chromatography, then extracted and analyzed using a nano-LC-MS/MS system composed of a united system composed of a TripleTOF 5600 mass spectrometer (AB SCIEX, Concord, ON, Canada) and liquid chromatography with cHiPLC nanoflex chip driven by a nanoUltra 2D Plus nano-LC (Eksigent, Dublin, CA, USA). Each sample was ran through a sampling course and a subsequent course of separation with tandem mass spectrometry (MS) analysis. The mode of tandem MS was information dependent acquisition. The resolving power of the screening performed by TOF MS was 30,000 full width at half maximum and the range of m/z was 350–1250 Th in 250 ms. When MS/MS was performed, the function of Enhance iTRAQ splitting and the auto-calculation of collision energy were launched.

ProteinPilot software 2012 (AB SCIEX, Framingham, MA, USA) was used to analyze the raw data file produced by MS scanning. The signaling of the iTRAQ114 group constituted the internal reference of the signal intensity and the normalized analysis was performed on all the signals. The proteomic database used in the present study was International Protein Index Human v3.87 fasta (http://www.ebi.ac.uk/, accessed on 30/11/2011; The European Bioinformatics Institute, 2013). Finally, the ratios of the proteins in the four groups with different iTRAQ tag labels were the averages of the ratios from two runs with the different labeling sequences mentioned previously.

### Immunohistochemistry assessment for the expression of S100A8 and S100A9 in different tissues

Paraffin blocks were sectioned 5 μm thick, mounted on slides and dried overnight. Sections were deparaffinized in xylene and rehydrated through decreasing graded alcohols. Subsequently, ethylene diamine tetraacetic acid buffer (pH 9.0) was used for antigen retrieval. Following incubation with 3% hydrogen peroxide solution to block the endogenous hydrogen peroxide enzyme, slides were immunostained with rabbit monoclonal antibodies for s100A9 (2738-1) and s100A8 (2732-1) (diluted in 5% BSA, 1:100), which were purchased from Epitomics Inc. (Burlingame, CA, USA), and the monoclonal rabbit horseradish peroxidase-conjugated secondary antibody (K5007; Dako, Glostrup, Denmark). Immune complexes were visualized by incubating with diaminobenzidine. The normal and cancer tissue samples of renal tissue from 18 cases in the R group were examined and compared with lesion samples from 18 cases in the C group and malignant lesions in the kidneys from 18 cases with TCC from the M group. Slides were reviewed and scored (% positivity and density) independently by two pathologists. Image pro-plus 6.0 (Media Cybernetics, Inc., Rockville, MD, USA) was used to analyze the images of the immunostained slides. Images were captured of three randomly-selected fields (magnification, ×200) for each slide in each group and the value of integrated optical density (IOD) for the positive areas with immunostaining in each image was calculated using the software. The higher value of IOD referred to the higher positive expression of the associated marker. The average of IOD from all the samples in each group was expressed as the mean ± standard deviation. Analysis of variance for significant differences in S100A8 and S100A9 among those groups was verified by SPSS 13.0 (SPSS, Inc., Chicago, IL, USA).

### Integrated analysis for the expression and function of the genes associated with S100A8 and S100A9 based on TCGA

TCGA is a project to catalogue genetic mutations and gene/microRNA expression/regulation associated with cancer, using recently developed high-throughput genome analysis techniques started in 2005. TCGA aims to provide genomic characterization and sequence analysis on >20 different tumor types in the following years. The gene/microRNA expression pattern and clinical information of >500 patients with kidney renal clear cell carcinoma (KIRC) were downloaded from the TCGA data portal (https://tcga-data.nci.nih.gov/tcga/). In the clinical database, there were 446 patients, of which, 71 patients have miRNA-sequence data from normal control kidney tissues and cancer tissues. The hierarchical clustering was performed for the expression of S100A8 and S100A9 proteins and their associated genes among RCC patients and normal controls from TCGA database. For profile clustering, the median expression value for each gene across the samples was set to zero. Cluster 3.0 and Tree View software (http://rana.lbl.gov/EisenSoftware.htm) was used for cluster analysis and representation (10). The hierarchical clustering was performed on genes and samples. Using a tree algorithm, these differentially expressed genes were organized based on similarities in the expression profile. This allowed visualization and selection of genes based on individual expression profiles.

In TCGA KIRC database, there were 31 normal controls, 197 T1, 49 T2, 162 T3 and 6 T4 samples. Based upon tumor grading (G1–G4), there were 31 normal controls, 5 G1, 173 G2, 169 G3 and 66 G4 samples in the database. The expression of S100A8 and S100A9 mRNA was investigated in different tumor stages and grades.

In the RNA-sequence database, there were 20,532 detected genes from 31 control and 419 KIRC samples (total 450 samples). Reads per kilobase per million mapped reads was used to normalize and quantify gene expression from RNA sequencing data. In the miRNA-sequence database, there were 1,216 detected micoRNAs in 71 normal controls and 497 KIRC samples (total 568 samples) and 243 were in the T1 stage. Reads per million mapped reads was used to normalize and quantify microRNA expression. The criteria to identify mRNA/microRNA function pairs involved the following conditions: i) Anti-correlation in expression (i.e. upregulated genes with downregulated microRNAs or down-regulated genes with upregulated microRNAs); ii) interaction between anti-correlated mRNA and microRNA predicted by TargetScan 6.0 and iii) enrichment analysis demonstrating that the number of mRNA targeted by their anti-correlated microRNA is significantly higher than the number of its targets in the whole human genome. Subsequently, the candidates of microRNAs identified in the present study were compared with the proteins S100A8 and S100A9 to determine the microRNA uniquely complementary to the structure of the genes S100A8 or S100A9.

## Results

### Proteins S100A8 and S100A9 are differentially expressed in serum among RCC patients and other controls, which is consistent with the results of the associated tissue gene expression

**Figure 1:**
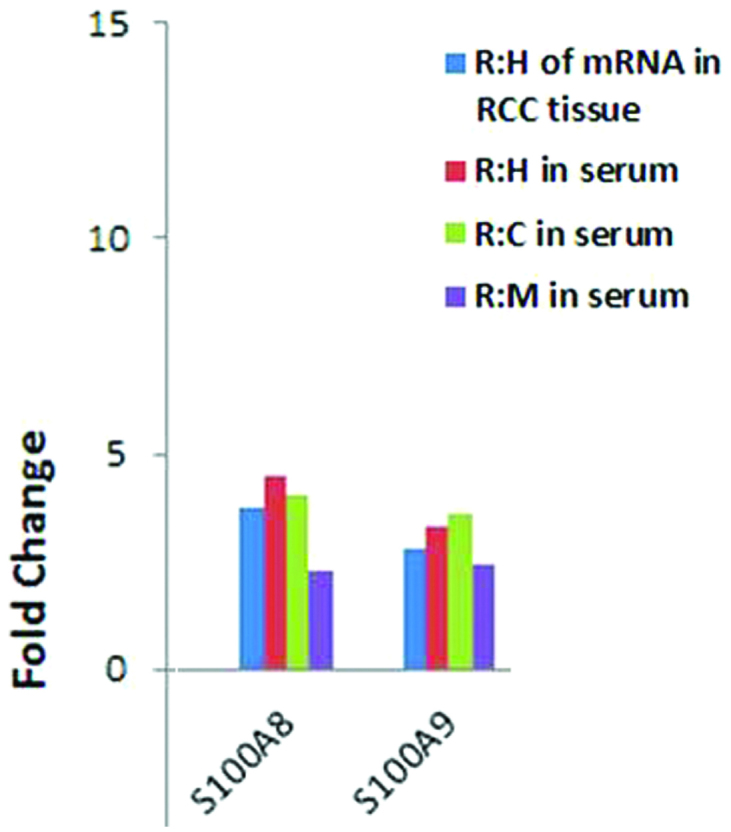
mRNA expression (P<0.01; fold change >2) and serum protein levels (fold change >1.5) of S100A8 and S100A9 changed consistently in RCC tissue. RCC, renal cell carcinoma; R, RCC cases; H, healthy controls; C, benign kidney lesion cases; M, other urological tumor cases.

A total of 29 patients with RCC in T1a stage, 24 with a benign renal mass, 28 with other urological tumors (18 with TCC) and 18 healthy controls were successfully enrolled in the present study (see the detailed characteristics of the cases in Table I). Through using cutoff values of 1.5-fold for overexpression and 0.67-fold for underexpression, the serum protein expression identified by two runs of iTRAQ analysis was compared in the RCC group with the benign lesions group, other urological tumors group and the non-cancer healthy kidney control group, respectively. S100A8 and S100A9 were upregulated in RCC patients compared with patients with benign kidney lesions, other urological tumors or non-cancer patients. Furthermore, the expression of S100A8 and S100A9 exhibited an upregulation in RCC compared with the normal control (Table II and Fig. 1).

### Immunohistochemistry results imply the differential expression of S100A8 and S100A9 in different types of tissue

**Figure 2:**
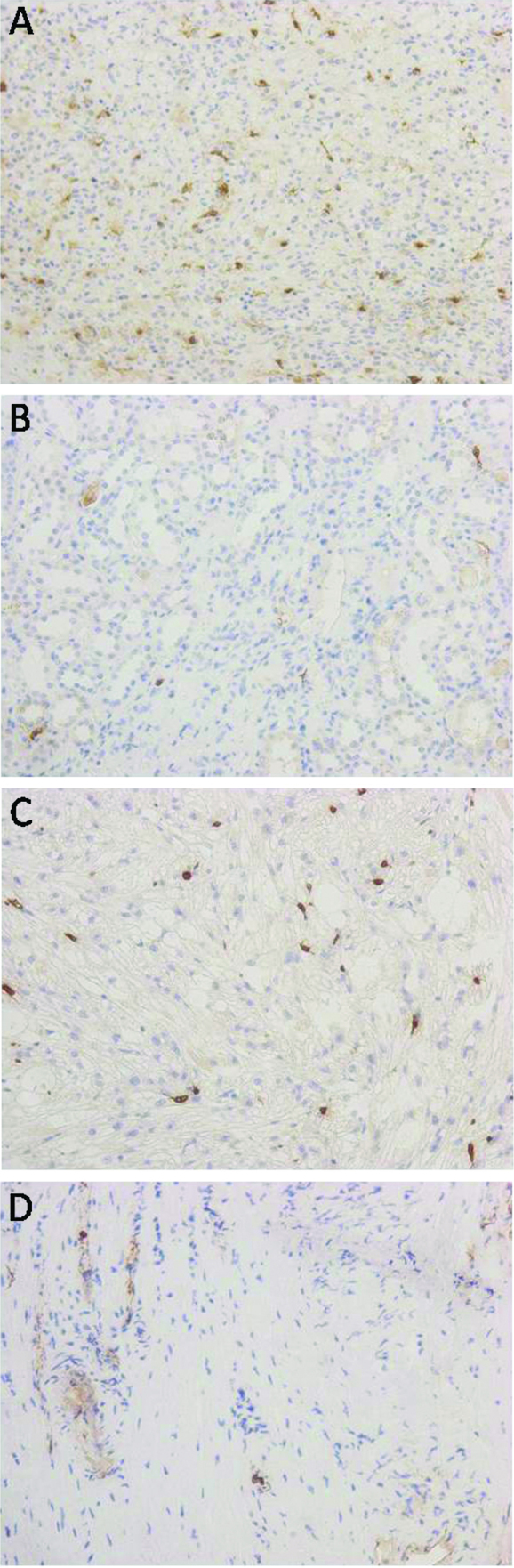
Immunohistochemistry results of S100A8 expression in different groups of tissue samples from (A) renal cell carcinoma, (B) normal kidney, (C) renal angioleiomyolipoma and (D) renal cyst.

**Figure 3:**
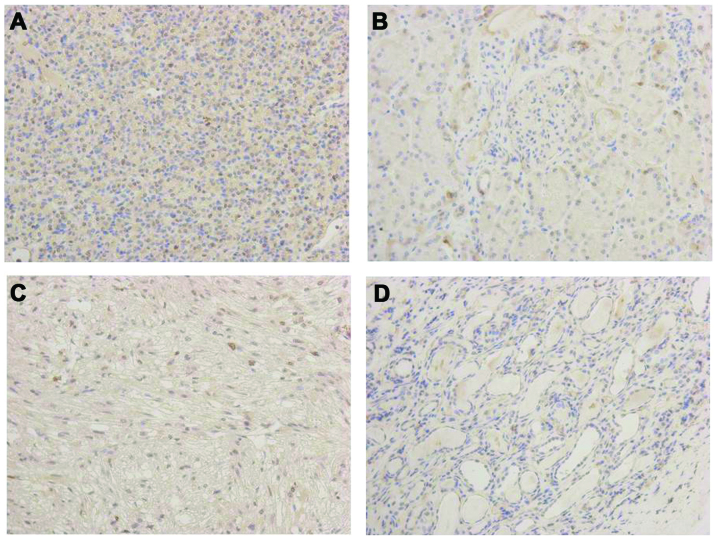
Immunohistochemistry results of S100A9 expression in different groups of tissue samples from (A) renal cell carcinoma, (B) normal kidney, (C) renal angioleiomyolipoma and (D) renal cyst.

The differential expression of S100A8 and S100A9 was validated among samples from RCC, normal kidney, benign lesions and TCC lesions in kidneys through immunohistochemical staining (Figs. 2 and 3). Furthermore, the mean IOD values for S100A8 and S100A9 in different types of tissues were calculated and analysis of variance was performed, presenting the statistical significance among different groups. Proteins S100A8 and S100A9 were upregulated in tumor tissues, which was consistent with that in the serum levels (Table III A and B).

### Cross-platform analysis using TCGA database demonstrates the differential expression of S100A8 and S100A9 between RCC and the controls

**Figure 4:**
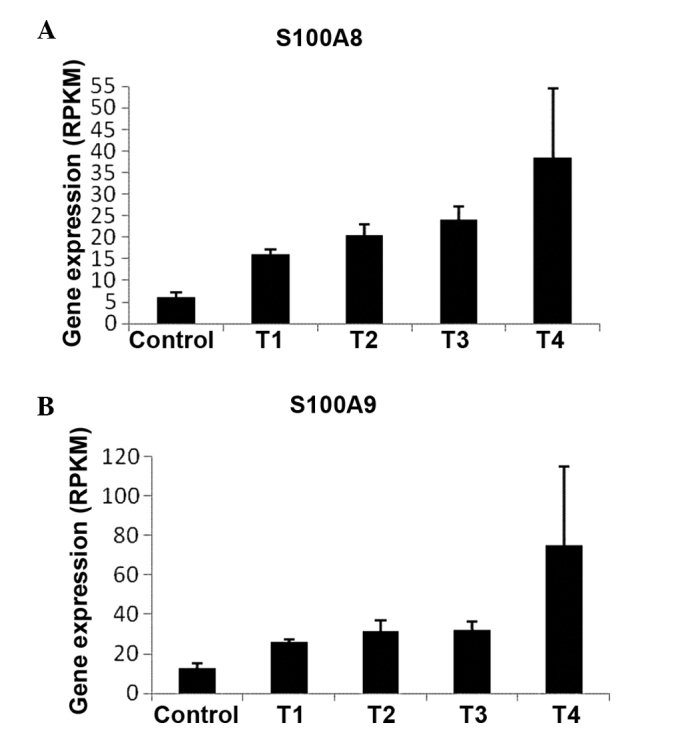
S100A8 and S100A9 are potential biomarkers for the identification of RCC tumor stages. Gene expression levels of (A) S100A8 and (B)S100A9 increased as the tumor stages of RCC advanced. RCC, renal cell carcinoma; RPKM, reads per kilobase per million mapped reads.

**Figure 5:**
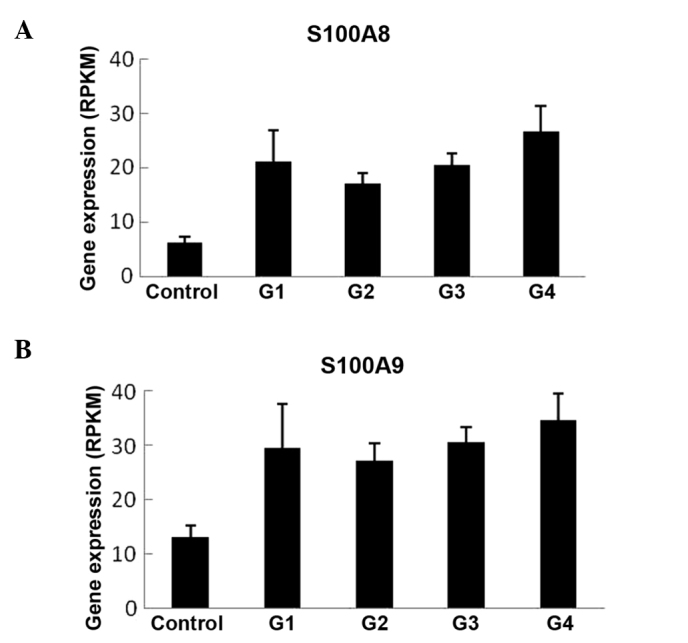
No significant differences were identified in the gene expression of (A) S100A8 and (B) S100A9 among different tumor grades. RPKM, reads per kilobase per million mapped reads.

The comparisons of S100A8 and S100A9 genes in normal control and different RCC tumor T stages indicated that with T stage progression, the upregulation of S100A8 and S100A9 genes increased (Fig. 4 and Table IV A). However, no significant difference was identified between the level of upregulation of S100A8 or S100A9 and the different tumor grades (Fig. 5 and Table IV B).

There were five microRNAs downregulated and potentially 298 upregulated mRNAs according to the microRNA/mRNA functional pair analysis (Table V). The Targetscan 6.0 was performed to identify whether the 3′ untranslated region (UTR) of S100A8 is complementary to the let-7 family nucleotide sequence. Let-7 had a confirmed decreasing expression in tumor tissues, while S100A8 level was upregulated. The data in Table VI showed the bioinformatics analysis of S100A8 and S100A9 using gene ontology, which presented that S100A8 and S100A9 were enriched in the process of defense response, inflammatory response and wound healing.

## Discussion

The S100 proteins are a multi-gene calcium-binding family comprising 20 known human members each coded by a separate gene. At least 16 of these genes cluster to chromosome 1q21, termed the epidermal differentiation complex (11,12). The S100 proteins belong to the Ca^2+^-binding EF-hand motif superfamily and have the ability to form homodimers. The C-terminal extension subsequent to the C-terminal EF-hand region and the hinge areas between the two EF-hand domains have the most variability between the different proteins and are therefore responsible for their specific biological properties (13). S100 proteins have a broad range of intracellular and extracellular functions. It has been demonstrated that they can interact with p53 (14), modulate cytoskeletal dynamics and cell proliferation, mediate metastasis of malignant tumors (15) and act as tumor promoters or suppressors (16). Therefore, there are currently numerous pieces of evidence implying that there is an association between those proteins and carcinogenesis with the formation of metastatic niches.

Compared with a number of other members of the S100 protein family, the expression levels of S100A8 and S100A9 in several types of tumor were significantly different to those in normal samples, but rarely in RCC. S100A8 and S100A9 expression levels increased in several types of cancer (10), including gastric, colon, pancreatic, bladder, ovarian, thyroid, breast, skin and prostate cancer (8,17–21). Previous studies have implied that S100A8 and S100A9 have a pathogenic effect in cancer progression in a concentration-dependent manner. At low concentrations, S100A8/A9 complexes promote tumor cell growth (9,22) and tumor cell migration (23), while at high concentrations, apoptotic effects on tumor cells were observed (22).

In the present study, S100A8 and S100A9 were demonstrated to be upregulated in RCC. They were identified as secreted proteins and involved in the defense response based on gene ontology analysis using the database for annotation, visualization and integrated discovery. Through TCGA analysis, it was determined that the mRNA levels of S100A8 and S100A9 genes were associated with RCC tumor T stage, which demonstrated that a higher expression indicated a higher stage. However, in the present study, S100A8 and S100A9 did not exhibit an association with RCC tumor grades. In addition, the differential expression of S100A8 and S100A9 was validated among samples from RCC, normal kidney, benign lesions and TCC lesions in kidneys through immnunohistochemical staining and the semiquantitative analysis of the IOD results from the stained slides.

To the best of our knowledge, the present study is the first to demonstrate the differential expression of S100A8/A9 and their associated genes among patients with RCC, benign renal mass, normal kidney and other types of urological malignant tumor at a serum and tissue level and is also the first to confirm the differential expression among the different types of cancer cell populations through transplatform bioinformatics analysis. Although S100A8 and S100A9 failed to reflect the differential grades of RCC, they remain promising biomarkers.

It is well established that S100A8 and S100A9 are abundant in the cytosol of phagocytes and are critical in numerous cellular processes, including motility and danger signaling by interacting and modulating the activity of target proteins (15). The data in Table VI showed the bioinformatics analysis of S100A8 and S100A9 using gene ontology, which presented that S100A8 and S100A9 were enriched in the process of defense response, inflammatory response and wound healing. S100A8 and S100A9 proteins can regulate the accumulation of myeloid-derived suppressor cells, the process, which leads to inhibition of dendritic cell differentiation and suppression of antitumor immune responses (16,24). It has been identified that primary tumors secrete soluble factors, including vascular endothelial growth factor A (VEGFA), transforming growth factor-β and tumor necrosis factor-α, which induce expression of S100A8 and S100A9 in myeloid and endothelial cells within the lung prior to tumor metastasis (25). S100A8 and S100A9 also increase the motility of circulating cancer cells by p38 mitogen-activated protein kinase (MAPK)-mediated activation of tumor cell pseudopodia (26). Furthermore, the expression of VEGF and p38 MAPK have already been associated with kidney cancer in previous studies (27,28). Therefore, future studies are required to further elucidate the molecular mechanisms underlying S100A8/A9-mediated carcinogenesis and metastasis of kidney cancer.

It has been identified that the 3′UTR of S100A8 is complementary to the let-7 family nucleotide sequence and thus microRNA let-7 is a potential target to focus on. MicroRNAs are noncoding RNAs that regulate numerous target genes through a post-transcriptional mechanism and thus control major developmental pathways. The phylogenetically conserved let-7 miRNA regulates cell proliferation and differentiation, thus functioning as a key regulator of developmental timing in *C. elegans* and a tumor suppressor gene in humans. Reduced let-7 expression in lung cancer may contribute to tumorigenic transformation through upregulation of these oncogenes and reduced let-7 expression levels are prognostic for poor patient survival rates. Let-7 has also been demonstrated to function as a tumor suppressor in breast cancer, where it controls proliferation and differentiation of tumor initiating cells.

In conclusion, the present data indicate that S100A8 and S100A9, which are upregulated in RCC, may serve as potential biomarkers for the detection of RCC or even promising targets for therapeutic intervention in RCC.

## Figures and Tables

**Table I:** Clinical characteristics of the selected patients.

Characteristic	RCC	Benign renal mass	Other urological tumors	Healthy control	Statistics	P-value
Number of cases	29	24	28	18		
Gender						
Male	20	12	26	16	14.92^a^	0.002
Female	9	12	2	2		
Age (years)						
Range	32–82	24–76	32–83	19–78		
Mean ± SD	52.83±13.54	54.29±13.33	63.75±13.70	45.06±19.20	6.272^b^	0.001

a K-value,

b F-value. P-value, significance of the gene expression change between normal control and RCC tissues. RCC, renal cell carcinoma; SD, standard deviation.

**Table II:** iTRAQ identified that serum proteins S100A8 and S100A9 were upregulated in T1a stage RCC, consistent with the tissue gene expression changes in the TCGA database.

IPI serial no.	Gene	Fold ratios for differentially expressed proteins			Normal control		T1 RCC		P-value	FC	Tissue genes	SP
		R:H	R:C	R:M	Mean	SD	Mean	SD				
IPI00007047	S100A8	4.4943	4.060075	2.269848	6.23674	6.064987	23.22495	34.21335	0.006364	3.723893	Up	Up
IPI00027462	S100A9	3.33155	3.610067	2.434098	13.1029	11.65109	36.49804	50.11201	0.010362	2.785493	Up	Up

P-value, significance of the change in IOD values among the R, C, M and H groups. R, T1a RCC; H, healthy person; C, benign mass lesions in kidney; M, other malignant urological tumor; SP, serum proteins; RCC, renal cell carcinoma; SD, standard deviation; iTRAQ, isobaric tags for relative and absolute quantification; FC, fold change; Up, upregulated.

**Table III:** IOD values of S100A8 and S100A9 from cases of RCC, renal cysts, renal hamartoma and normal kidneys.

A, IOD values of S100A8				
Group	n	Mean IOD ± SD for S100A8	Fold	P-value
Renal cyst	9	698.31±298.01^a^	5.572	0.002
Renal hamartoma	9	745.14±229.71^b^		
Normal kidney	15	314.67±148.06^c^		
Renal cell carcinoma	16	697.06±445.88		

B, IOD values of S100A9				

Group	n	Mean IOD ± SD for S100A9	Fold	P-value

Renal cyst	9	1460.48±1113.20^d^	8.76	0.000
Renal hamartoma	9	3408.81±1648.36^e^		
Normal kidney	15	827.82±562.90^f^		
Renal cell carcinoma	18	2641.61±1724.36		

a LSD test revealed that the significance of the difference in mean IOD values between renal cyst and RCC was 0.992.

b LSD test revealed that the significance of the difference in mean IOD values between renal hamartoma and RCC was 0.715.

c LSD test revealed that the significance of the difference in mean IOD values between normal kidney and RCC was 0.001.

d LSD test revealed that the significance of the difference in mean IOD values between renal cyst and RCC was 0.038.

e LSD test revealed that the significance of the difference in mean IOD values between renal hamartoma and RCC was 0.173.

f LSD test revealed that the significance of the difference in mean IOD values between normal kidney and RCC was 0.000. P-value, significance of the change in gene expression between normal control and RCC tissues. IOD, integrated optical density; RCC, renal cell carcinoma; SD, standard deviation; LSD, least significant difference; IHC, immunohistochemistry.

**Table IV:** Comparisons of the relevant genes in normal control and different RCC tumor T stages and tumor grades.

A, Comparisons of the relevant genes in normal control and different RCC tumor T stages										
Gene	Control	SEM	T1	SEM	T2	SEM	T3	SEM	T4	SEM
S100A8	6.23674011	1.089303788	16.14074787	1.062754347	20.39068	2.683801894	24.02628	3.193372	38.48324	16.11524533
S100A9	13.10290141	2.092596937	25.81050782	1.73274309	31.82967	4.97714547	32.41733	3.928143	75.03031	39.9230471

B, Comparisons of the relevant genes in normal control and different RCC tumor grades										

Gene	Control	SEM	G1	SEM	G2	SEM	G3	SEM	G4	SEM

S100A8	6.23674011	1.089303788	21.11229	5.676431387	17.09285789	1.887268	20.40151	2.229353	26.69588	4.571362
S100A9	13.10290141	2.092596937	29.41922	8.039944646	27.14093336	3.218097	30.52059	2.796429	34.51957	4.959358

SEM, standard error of the mean; RCC, renal cell carcinoma.

**Table V:** Five downregulated microRNAs identified using microRNA/mRNA functional pair analysis.

Category	MicroRNA	Count in selected genes	Count in total pop.	P-value
TargetScan6.0	let-7	90	1073	0.013609875
TargetScan6.0	miR-138/138ab	51	560	0.014336931
TargetScan6.0	miR-30bc	110	1358	0.017904491
TargetScan6.0	miR-485-5p	34	379	0.047137237
TargetScan6.0	miR-125b-5p	69	848	0.048097443

Count in selected genes: The number of upregulated genes targeted by this microRNA; count in total population: the number of genes in the human genome targeted by this microRNA.

**Table VI:** Gene ontology (biological process) analysis for S100A8 and S100A9.

Category	Term	%	P-value	Fold enrichment	FDR
GOTERM_BP	Defense response	40.74074	1.38E-08	10.52018381	2.07E-05
GOTERM_BP	Inflammatory response	37.03704	9.02E-10	18.09765886	1.35E-06
GOTERM_BP	Response to wounding	37.03704	6.41E-08	11.097621	9.6E-05

The National Institutes of Health Database for Annotation, Visualization and Integrated Discovery indicated that the differentially expressed serum proteins S100A8 and S100A9 in renal cell carcinoma predominantly possess inflammatory, defensive and wound healing functions. FDR, false discovery rate.
